# Modulation of cerebral haemodynamic response to olfactory stimuli by emotional valence detected by functional magnetic resonance imaging

**DOI:** 10.1590/1980-57642015DN94000405

**Published:** 2015

**Authors:** Cristofer André Caous, Patrícia Renovato Tobo, Vânia Hercília Talarico, Luciana Ribeiro Lopes Gonçales, Elise Yoshimine, Antonio Cesário da Cruz Jr, Cristóvão Albuquerque, Edson Amaro Jr

**Affiliations:** 1 Instituto do Cérebro, Instituto Israelita de Ensino e Pesquisa Albert Einstein, São Paulo SP, Brazil.; 2 Natura Inovação Tecnologia Produto Ltda, Cajamar SP, Brazil.; 3 NIF/LIM44, Department of Radiology, Hospital das Clínicas, University of São Paulo SP, Brazil.

**Keywords:** BOLD - blood oxygenation level dependent, cerebral cortex, emotion, fMRI, human olfactory function, odorant, BOLD - blood oxygenation level dependent, cortex cerebral, emoção, RMf, olfato, odorantes

## Abstract

Olfactory perception, although restricted to just a few contexts in everyday life, is key in medicine. Several dementia conditions have been associated with early loss of olfactory discrimination. Despite the fact that several brain areas have been associated with olfaction in functional magnetic resonance imaging (fMRI), the mechanisms by which emotional valence is conveyed to the brain are not fully understood.

**Methods:**

In this study, we compared cerebral activations by olfactory stimuli using different emotional valence stimuli on event-related fMRI. We used three standard olfactory odorants with different valence (positive, neutral and negative). Forty-three healthy subjects (22 males) were scanned on a 3.0T MR system. Olfactory stimulation was attained through a delivery system synchronized with image acquisition and subjects´ breathing instructions. fMRI data analysis was performed by the FSL package (Oxford University) including head movement correction, GLM modeling of the neurovascular (BOLD) response and group activation maps produced at p<0.05and corrected for multiple comparison.

**Results:**

Increased cerebral responses within the anterior cingulate, amygdaloid nuclei, as well as the dorsolateral prefrontal, occipital and orbitofrontal cortices were observed in positive and negative valence conditions, while response to neutral valence arousal was less intense and not observed in the amygdaloid complex. The most significant statistical response aroused from the stimuli clusters was observed in the negative condition.

**Conclusion:**

The results of the present study support the hypothesis that neutral stimuli may be more sensitive to early losses in pathological conditions, particularly dementia.

## Abstract (pt)

Apesar de praticamente restrita a alguns contextos na rotina diária, a percepção de estímulos olfativos é bastante relevante em medicina. Várias condições patológicas, e em particular as demências, estão associadas à perda precoce da capacidade de discriminação e percepção olfativa. Apesar de estudos de ressonância magnética funcional (RMf), terem identificado várias áreas cerebrais relacionadas à percepção olfativa, ainda não sabemos quais destas são moduladas pela valência emocional do estímulo olfativo. Neste estudo investigamos o padrão de resposta cerebral à odorantes com diferentes valências emocionais em indivíduos saudáveis estudados por RMf relacionada à eventos.

**Métodos:**

Comparamos a resposta cerebral em um desenho de RMf relacionado à eventos e com uso de três estímulos olfatórios padronizados e com diferentes valências emocionais (positiva, neutra e negativa). Quarenta e três indivíduos normais (22 homens) foram estudados por RMf relacionada à eventos em aparelho de 3T. A estimulação olfativa foi realizada por meio de sistema sincronizado à aquisição das imagens e às instruções dadas aos sujeitos. A análise dos dados foi feita por meio do software FSL (Universidade de Oxford) e incluiu correções de movimento, modelagem de sinal com técnica GLM e mapas de grupo com nível de significância < 0,5.

**Resultados:**

As respostas cerebrais no cíngulo anterior, bem como no córtex dorsolateral pré-frontal, occipital e órbitofrontal foram observadas nas condições de valência positiva e negativa, porém a resposta ao estímulo neutro foi menos intensa e não foi observada em complexos amigdaloides. A resposta estatística mais significante foi observada no cluster de estímulos com valência negativa.

**Conclusão:**

Estes dados representam evidências para a hipótese de que possivelmente a perda da percepção discriminativa de estímulos com valência emocional neutra pode ser mais sensível em relação às manifestações iniciais de condições patológicas, inclusive demências.

## INTRODUCTION

Neurobiological interpretation of emotional stimuli relies on the personal significance or experience of a given stimulus, a response-level of reaction related with an affective state and how this affective state is regulated.^1^ The chemical sense of smell is different from the other senses because it may have different functional lateralization depending on the nature of the stimuli and intrinsic judgment related with pleasant or unpleasant experiences.^2^

How the brain interprets odorant-induced stimulation depends on the neural topographical organization of the brain itself. Although the olfactory system and the different physiological mechanisms it may trigger have been extensively studied, central nervous system processing of odorant-related stimulation is not yet fully understood.

Briefly, earlier investigations have described the interpretation of odorant-induced stimulus through three distinct pathways that comprise olfactory, trigeminal, and vomeronasal elements. The piriform cortex is considered the primary olfactory site activated by perception of any subtle odorant.^3,4^ The neuronal activity level of the piriform cortex appears to be modulated according to a specific task or condition when the individual is submitted to certain odorant-induced stimuli.^5^ Odorants are capable of impacting a neurological decision or judgment. Again, depending on the nature of the stimulus, individuals may experience sensations of pleasure, acquaintance, and intensity, among others.

Neuroimaging data have revealed a neuronal interaction between a negative emotional state and the level of olfactory acuity occurring in the primary olfactory circuitry.^6^ In fact, it is known that individuals have a well-defined decision criterion regarding one particular odorant over another.^7^ This makes dissociation between a chemical and therapeutically odorant-induced effect very difficult. When a scent is inhaled or perceived, a cognitive response is triggered according to the individual's olfactory memory.

Based on the olfactory memory, the individual decides whether or not the scent is potentially harmful. Previous findings strongly suggest that both neural-dependent and non-neural-dependent mechanisms occur in olfactory system comprehension.^8,9,10^ Physiological responses of olfactory perception have also been associated with emotion and personality. It has been observed that anxious individuals show a faster reaction to a given emotional valence compared to response to a neutral odorant stimulus.^11^ The transmission of the sensitive olfactory input occurs immediately after the primary neuronal activation at the upper region of the vomeronasal neuroepithelium.^12^ The somatosensitive component related with the trigeminal system and olfactory sensations are essential for olfactory acuity.^13,14^ In addition, previous results indicate that a high neuronal activity within the amygdala responds to aversive emotional arousal in order to prepare a biological response to a given external stimulus, regardless of the stimulus valence.^15,16,17^

It has been suggested that patients with Parkinson's disease present different cortical and subcortical activation patterns to those observed in control groups.^18,19^ Dysfunctional working circuitries in early Parkinson's disease have been studied pertaining to neuronal plasticity and hyperactivation. These cellular events occur when plasticity and hyperactivation attempt to restore brain function. This suggests that a better understanding of olfactory dysfunction in early premotor Parkinson's disease might be enlightening.^20^ In other neurodegenerative diseases, such as Alzheimer's disease, important findings such as the presence of neuritic dystrophy in the olfactory epithelium indicate that a close relationship exists between neurites and olfactory cellular injury. Additionally, psychophysical data reveal that many schizophrenia patients have different levels of olfactory dysfunction, which are believed to have a genetic component.^21^

A challenging idea is to establish many neurodegenerative or psychological conditions associated with olfactory system function, defining the neuroanatomical and biochemical substrates involved. In the present study, the smelling sense was previously tested with a set of stimuli, as pleasant or positive, neutral, and unpleasant or negative stimuli activate specific neural pathways in the brain. Do the tested odorants have a similar olfactory brain response regarding emotional valence or BOLD-effect intensity when comparing negative, neutral and positive stimulation? Functional magnetic resonance imaging (fMRI) was used to investigate hemodynamic activity in the human brain and identify potential components allowing the formulation of new hypothesis, rather than finding answers. The present study focused on olfactory brain activity related with distinct BOLD-effect patterns. The olfactory paradigm was designed to analyze brain response to single odorants with clear emotional components, allowing the relationship between emotional valence and hemodynamic response of the brain to be studied.

In this study, chemosensitive induction with odorants was applied in a cross-modal delineation comparison in which distinct emotional valence responses could be detected and analyzed. The aim was to monitor the valence perception network in a healthy sample recruited from the local population. This approach may also help in the study of prodromal phases of neurodegenerative diseases in which the olfactory system is affected early.

## METHODS

**Ethics.** Ethical approval was provided by the Research Ethics Committee of Albert Einstein Hospital, São Paulo, Brazil. The present investigation was conducted in accordance with the Declaration of Helsinki, as adopted and promulgated by the United States National Institutes of Health. Written informed consent was obtained from all participants after a detailed explanation of the study.

**fMRI activation task.** Subjects participating in this study also participated in other tasks, the results of which are being prepared for submission elsewhere. All subjects performed an olfactory event-related fMRI task as detailed below.

After subjects had undergone a standardized psychophysical olfactory test, three different odorants were presented to the participants: one considered negative or unpleasant, another positive or pleasant, and a neutral representative odorant. Each olfactory stimulus was delivered in 15 trials to each participant. Each trial consisted of 2s stimulus delivery followed by 12s of continuous ambient airflow. Olfactory induction was achieved by odorant stimulation delivered to the subject's nose. In order to synchronize individual breathing rate, subjects were instructed to inhale normally and were cued by a visual stimulus indicating olfactory stimulation. A cross was continuously displayed center screen, which was replaced by a circle 2s before stimulus delivery. Subjects viewed the visual cue via a head-coil mounted mirror. Occasionally, breathing pace did not follow the stimuli-attention applied paradigm, as breathing rhythm can be controlled voluntarily. Subjects were instructed to rate the stimuli as good or positive, neutral, or bad or negative through a push button system as soon as the stimuli were perceived.

**Subjects.** A total of 43 healthy volunteers (22 males; mean age 29.4±7.5years) were studied in the present investigation. Participants were excluded if any of the following conditions were present: pregnancy, traces of anxiety, nasal polyposis, sinonasal disease, allergic rhinitis, hyposmia, post-traumatic olfactory loss, laryngitis, pharyngitis, headache of any kind, smoking or alcohol addiction, depression and psychosis or any neurological condition diagnosed in a structured interview applied by the investigators. Self-evaluation questionnaires (Beck Depression Inventory, VAMS and IDATE) were also used to screen for outlier performance. Six of the subjects in the sample were left-handed according to the Laterality index questionnaire.^22^ All subjects were trained on the entire experimental procedure before the MRI sessions. All subjects were exposed to the six odors during the testing sessions and these included the three stimuli used during image acquisition sessions (see below).

**Pre-scanning olfactory tests.** The subjects were submitted to a Sniffin' Sticks adapted test^23^ in order to evaluate olfactory function. Intensity (soft or intense) and emotional valence features (pleasant or unpleasant) were considered and measured on a scale from 1 to 10 for each of the six odorant samples. Samples comprised rosemary, sandalwood, vanillin, lavender, and AIN-fish oils and dipropylene glycol solution (International Flavor & Fragrances - IFF, São Paulo, Brazil). The latter three samples were applied in the olfactory fMRI paradigm, considered as pleasant or positive, very unpleasant or negative, and neutral odorants by all participants.

**Odorant stimulation during fMRI.** Olfactory odorant induction was attained through a delivery system^24,25^ constituted by high performance air valves connected to Teflon tubes in order to deliver the stimuli to the subject's nose. Each Teflon tube was attached to a specific reservoir via a handmade Teflon adapter, which allowed any odorant to be released automatically at a given instant programmed in the computer with the corresponding designed paradigm. It is important to note that several odorants were pre-tested because careful consideration was given to their trigeminal properties in olfactory assessment. Three odorants, one pleasant, one neutral, and one unpleasant were used. The pleasant odorant was lavender oil 10%; the unpleasant was AIN-fish 1%; and the neutral odorant consisted of a dipropylene glycol solution (International Flavor & Fragrances - IFF, São Paulo, Brazil). They were selected and formulated to be liquid at room temperature.

**fMRI data acquisition.** The images were acquired with a 3.0T Magnetom Trio (Siemens^®^, Erlangen, Germany) scanner within the Magnetic Resonance Imaging Department at Albert Einstein Hospital, where T2-weighted EPI slices were acquired every 30 s (TR=2000 ms). Axial slices (32) with 3.3mm isotropic voxels and between-plane spacing of 1.0 mm were used. Each run comprised 315 volumes in total (t=10m12s). Three RF pulses were applied before each EPI-BOLD acquisition in order to avoid T1 saturation effects.

**fMRI statistical analysis.** Imaging data was analyzed using FSL software (Oxford Centre for Functional MRI of the Brain - FMRIB Analysis Group, Oxford University, United Kingdom). Pre-processing included motion correction, realignment, non-encephalic tissue removal, and voxel time series smoothing with a 5 mm Gaussian FWHM filter (Gaussian-weighted LSF straight line fitting, with δ=50 s). The fMRI imaging studies are based on statistical comparisons among blood oxygen level-dependency signaling (BOLD) task-related control conditions. The statistical time series analysis was based on the FILM autocorrelation correction algorithm.^26^ Images were cluster-limiarized Z>3.0and significance level of *p* was 0.05.^27^ Group maps representing each emotional valence condition were produced by FLIRT algorithm.^28,29^

Ratings of odorants for the stimuli were acquired throughout the scanning process upon each trial for all the healthy control subjects.

## RESULTS

**Behavioural analysis.** After the fMRI acquisition, some participants judged the olfactory perception task "challenging" and others as "very easy". The congruence rate was low: 49% of the answers to the olfactory stimuli correctly distinguished or categorized the stimuli according to mean group response. The analysis of the behavior data (button responses) during the olfactory fMRI paradigm showed 45% correct answers for positive or pleasant odorant, 30% for neutral, and 35% for negative or unpleasant odorants. All congruence rates were obtained individually and correctness of classification was based on comparison with the original database.

**fMRI Data.** All conditions produced responses in the insula, cerebellum, and pre-frontal and lateral orbitofrontal cortices (parts of the primary olfactory cortex).

The group map representing brain response in the negative condition showed BOLD response in the right parahippocampal gyrus, bilateral amygdaloid complex, primary visual cortices, anterior cingulate, posterior middle right frontal gyri and orbitofrontal cortex. Other areas involved included the bilateral superior cerebellar lobules, thalami, and right supramarginal gyrus compared to baseline.

The group map representing brain response in the neutral condition showed BOLD response in the inferior frontal gyri, bilateral dorsolateral prefrontal cortex, and in the superior parietal area. No response was observed in the amygdaloid complex, thalami or anterior cingulate gyrus.

Finally, the group map representing brain response in the negative condition showed BOLD response in the left insula, bilateral frontal-mesial lobe, inferior frontal gyrus, left dorsolateral prefrontal cortex, amygdaloid complex, and anterior cingulate gyrus.

## DISCUSSION

The BOLD effect response was observed in all expected areas for each emotional valence. The role of emotion in olfactory processing and faster behavioral reactions has been observed in anxious individuals and are attributed to specific emotional valence odors despite a non-concurrent approach.^11^

Regarding neuroanatomical structures and neuronal activation sites, some studies have shown that the amygdala plays a key role in decision mechanisms, irrespective of whether the individual's preference is required during a given task.^30^ The main finding of the present study was that these neural physiological events reveal that the amygdala and the regions within the orbitofrontal cortex (lateral and medial) have differential involvement in a neural system underlying the assortment of objectives based on the probable motivation value of defined stimuli, as explored herein. Lateral orbitofrontal cortex activity was found to be discriminatory when individuals had to choose among a few pleasant options or which substance or thing was their favorite.^31^ In addition, the medial orbitofrontal cortex is a region implicated in representing stimulus-reward patterns.^32^ According to other brain imaging studies, olfactory stimulus is handled identically to other sensory stimuli, recruiting the limbic complex neural system.^33^ For instance, previous observations have indicated that these areas are related to both visual and auditory brain processing. Other brain areas are claimed to be activated under olfactory stimulation, such as the more inferior parts of the anterior cingulate, temporal and occipital lobes, with butyric acid or vanilla inhalation. No significant difference was noted by these investigations in the amygdaloid body or in the entorhinal cortex. An event-related fMRI-design experiment study consisting of semantic modulation suggests that the affective value of the test odor is significantly more unpleasant when labeled with *body odor* than when classified as *cheddar cheese* in the odorant trial. In this case, activation of the rostral anterior cingulate cortex and medial orbitofrontal cortex are associated to pleasantness ratings. This cognitive modulation was also perceived for the odorant test in the amygdala bilaterally.^34^ Likewise, blind people in general have a lower threshold for detecting odors and fMRI results suggest a possible neuronal plasticity in the visual cortex area additionally to the other brain areas involved in olfactory processing. These activated areas in the cases reported were very strong in bilateral hippocampus, right orbitofrontal cortex, and amygdala.^35,36^

Interactions involving pleasant and unpleasant stimuli in a mixture of odors promote an attentional capture outcome mainly in the superior frontal gyrus despite unequivocal attentional arousal.^37,38^ However, a stimulus does not necessarily produce pronounced brain activity behavior, but an association between the BOLD-effect and behavior processing within brain areas occurs in many cases. It simply seems to be the same physiological path but is not. The emotional verdict related with the hemodynamic response was observed in the left neural hemispheric system, independently of whether emotions are induced via olfactory, visual, or auditory sensory stimuli.^8,39^ However, there are constraints to this kind of evidence regarding hedonic judgment valence activity.^40^ In the present study, neural substrates triggered by hedonic valence could not be clearly distinguished since pleasant and non-pleasant odorants were interrelated during the same scan cycle. Moreover the valence and intensity of the human olfactory system are claimed to have different neural substrates, where amygdala activation is implicated but does not actually respond to the valence *per se*.^40,41^ In manipulating the three odorants, a higher response was observed in the amygdala or medial orbital cortex for the AIN-fish when all odorants were equivalent for emotional valence.

The greater activation of the left amygdala to unpleasant odorants probably suggests the strength of a given emotional reaction. In a hazardous circumstance experience, where people inhale unpleasant or aversive odorants, natural reactions such as forced breathing saving and tension occur. The amygdala is considered an emotional processing site that receives and responds to stimuli originating from within the body and may have some level of physiological responsiveness in respiratory and autonomic mechanisms.^42^

Encephalic regions sensitive to behavioral modulation components of disgust faces identified a possible path facilitation effect when compared to an odorant. The regulation of underlying visual modulation of olfactory perception demonstrated that the sense of smell is much faster when odorants are inhaled in the context of corresponding visual signs. This kind of behavioral effect was observed in the anterior hippocampus and orbitofrontal regions.^43^ Also, the neuronal modulation process encompassed the anterior insula (chemical senses) and the fusiform gyrus activated ROIs.^44^ This physiological function may be explained by the fact that this brain area works like a relay, receiving neuronal inputs from somatosensory stimuli and presenting end-outputs for the sensory network.^45^ Thus, central emotional responses to different odorants are recruited to support emotion displayed-avoidance behaviors, and cross-modal analyses are very useful for evaluating the chemosensory stimuli of odorants.

The insula and the orbitofrontal cortex were constantly activated during the emotional and odorant valence neuronal processing in our data. This result is in agreement with previous positron emission tomography observations showing that single familiarity judgments do not involve the olfactory component (only visual representations), activating the right medial orbitofrontal cortex. This same area was bilaterally triggered using odorants with strong affective valence.^8,42^ The orbitofrontal cortex is not completely developed in rodents but is very well developed in primates. Visual inputs reach the orbitofrontal cortex from the inferior temporal cortex, temporal sulcus, and pole. Its caudal extent receives neuronal inputs from the amygdala whereas other subregions have strong inputs from the nucleus of the thalamus. Neuroimaging studies have identified the orbitofrontal cortex as a main target for emotion processing, representing many reward or punishment situations encompassing human behavior responses.^46,47^

An important clinical application of olfactory fMRI tests is for early detection of patients at risk for Parkinson's and Alzheimer's diseases. In fact, Wang et al. showed reduced brain activation in a group of patients with Alzheimer's Disease compared to age-matched controls using fMRI.^48^ However, the authors did not use olfactory stimuli with different emotional valence levels and the fMRI study employed a block-design, thus not allowing the subject to inform how they perceived each individual stimulus delivered. Nevertheless, all areas involved in the olfactory perception network detected in the control group of the study were also observed in the present study, except for the amygdaloid complex. This fact may be due to the above-mentioned methodological differences, but it is likely that participant age difference (present sample was younger) may also account for this difference, since it is known that elders have lower magnitude fMRI responses.^49^ On the other hand, it is known that subjects at risk for dementia may already have alterations in brain response. Bookheimer et al.^50^ compared healthy older persons who were ApoE3 and ApoE4 carriers, showing that individuals carrying the ApoE4 allele already showed differences in brain response on an fMRI memory task, despite exhibiting normal performance on neuropsychological tests.^50^ However, to act in prevention, the target population should be younger, as signs of brain alterations in dementia occur earlier in life. In this context, our study suggests that using olfactory tests, especially those without negative content, may be an even more sensitive method for detecting alterations in the population at risk. However, a cautionary note must be made: individual fMRI tests are known for their high variability and thus normative databases and careful test designs are required before introduction into clinical settings.^51^

In conclusion, distinct olfactory sensory stimuli comprising neutral, positive and negative emotional valence engaged the cerebral olfactory network with different modulation in key structures, notably the anterior cingulate, frontal cortex and insula. The most significant statistical response aroused from the stimuli clusters was observed in the following order: neutral, positive and negative. The present hypothesis-generating study contributes information on how pleasant and unpleasant stimuli are processed in normal volunteers. This data can be used for planning test-bed investigations of new therapeutics using olfactory emotional neuronal system as a proxy in the prodromal phase among patients at risk for dementia.

## Figures and Tables

**Figure 1:**
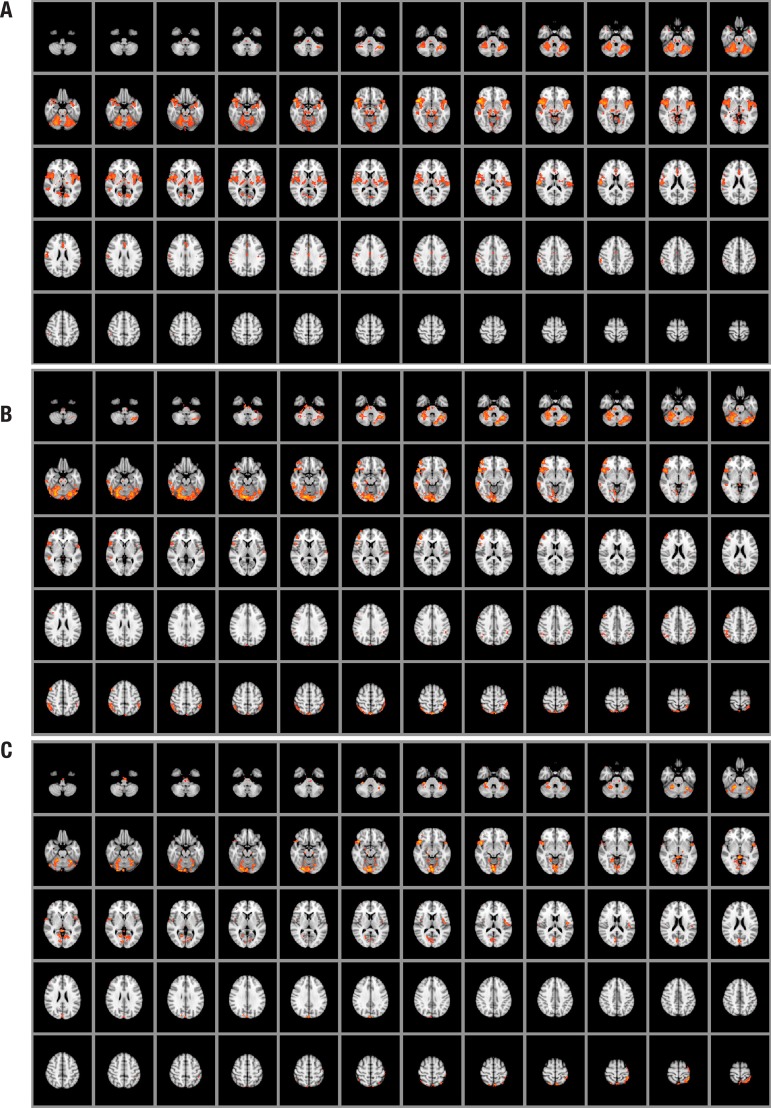
Brain areas with activation (BOLD effect) for olfactory pre-defined stimuli of negative [A], neutral [B], and positive [C] odorant inhalation. In [A], showing amygdaloid complex, the right parahippocampal gyrus, right primary visual cortex, and orbitofrontal cortex; in [B], the inferior frontal gyri, bilateral dorsolateral prefrontal cortex, and in the parietal area the precuneus were observed. In [C], the left insula cortex, bilateral frontal-mesial lobe, inferior frontal gyrus, left dorsolateral prefrontal cortex, and anterior cingulate are shown (each condition is shown in 60 2D axial images oriented in the AC-PC plane, slices spaced by 2 mm in the inferior-superior direction, starting from MNI Z-40 mm to Z+36 mm; cluster-wise p-value < 0.05; Z >3.0).

**Figure 2:**
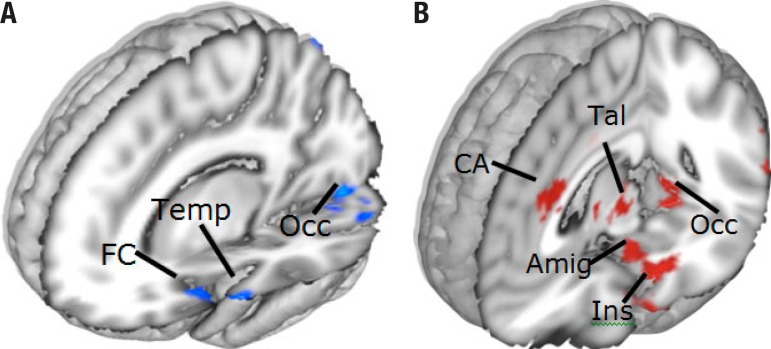
Brain areas rendered in 3D maps showing different aspects of emotional olfactory response. Panel [A] depicts the olfactory stimuli that were all pleasant or with a positive emotional valence in blue. Panel [B] depicts the responses of the same group of participants to unpleasant or negative emotional valence in red. CA: anterior cingulate; FC: frontal cortex; Ins: insula; Occ: occipital lobe; Par: parietal lobe; Tal: thalamus; Temp: temporal lobe; Amig: amygdaloid complex.
